# The gut bacterial communities across six grasshopper species from a coastal tallgrass prairie

**DOI:** 10.1371/journal.pone.0228406

**Published:** 2020-01-30

**Authors:** Melani Muratore, Chelse Prather, Yvonne Sun

**Affiliations:** Department of Biology, University of Dayton, Dayton, Ohio, United States of America; University of Maine, UNITED STATES

## Abstract

Insect microbiomes play an important role in the health and fitness of insect hosts by contributing to nutrient absorption, immune health, and overall ecological fitness. As such, research interests in insect microbiomes have focused on agriculturally and industrially important organisms such as honey bees and termites. Orthopterans, on the other hand, have not been well explored for their resident microbial communities. Grasshoppers are an integral part of grassland ecosystems and provide important ecosystem services. Conversely, grasshoppers can be an agricultural pest requiring management with broad spectrum pesticides. However, little is known about the microbiomes of grasshoppers and their potential contribution to grasshopper biology. Here we examine the gut microbiome of six species of grasshoppers (n = 60) from a coastal tallgrass prairie ecosystem to gain a better understanding of the microbial communities present across the orthopteran order in this ecosystem. We found that there are bacterial phyla common to all six grasshopper species: Actinobacteria, Proteobacteria, Firmicutes, and to a lesser degree, Tenericutes. Although the grasshopper species shared a high relative abundance of these groups, there were notable shifts in dominant phyla depending on the grasshopper species. Moreover, measures of alpha diversity revealed a more diverse microbiome in males than females. Our observations support the hypothesis that there is a “core” group of bacterial families in these grasshopper species and factors such as trophic behaviors and the evolution of the host may contribute to the shifts in prevalence among these core microbial groups.

## Introduction

The relationships between insect hosts and their microbiomes have wide-ranging effects on the host, and by extension, the ecosystem. For example, termites, wood-feeding cockroaches, and carrion-feeding burying beetles are aided by endosymbionts in nutrient acquisition [1,2]. Female red firebugs of the genus *Pyrrhocoris* smear their eggs with their microbiota in order to ensure the success of their offspring [3]. Many lepidopterans also transfer microbes vertically by smearing their microbiota on the eggs after oviposition [4]. Other host-microbiome effects include defending the host against exogenous microorganisms that may be pathogenic [5] and influencing host behaviors like aggregation into large groups [6]. With the great diversity of insect taxa, our understanding of the extent their microbiomes contribute is still emerging.

Orthopterans, including both short-horned and long-horned grasshoppers, are key herbivores in worldwide grassland ecosystems and are one of the most important insect groups ecologically and economically [7]. Grasshoppers vary in their feeding habits. Some are strict herbivores, including specialist grass- or forb-feeders or generalists that feed on a wide range of plants. There are also omnivorous grasshoppers that are known to opportunistically feed on other insects or carrion. Because of their consumption of large amounts of plant biomass, grasshoppers function as key consumers that can increase the pace of nutrient cycling, serving as a food source for higher trophic level organisms like avian predators [8,9] and affecting both above- and belowground decomposition and soil enzyme activity [10–12]. As a result, grasshoppers have been suggested as a key indicator of the level of stability in grassland ecosystems, especially in threatened habitats such as prairies, where the soil fertility inevitably leads to agricultural use [13]. Alternatively, as a key consumer of plant mass, grasshoppers are also associated with 1.25 billion dollars of damage per year in the United States alone, inflicting crop damage and degrading range forage, often necessitating management with pesticides that create additional ecological disturbances [14]. Therefore, the ecological and the economic importance of grasshoppers necessitate a better understanding of their biology—including their microbial communities.

Much work has been done to characterize and understand the function of microbiomes in animal systems, with an increasing range of host models. The lion’s share of research on insect-herbivore microbiomes has focused mostly on model orders like the hymenopterans (including the honeybee), which are highly charismatic and offer obvious ecosystem services [15–17]. Although orthopterans are ecologically and economically significant as a group, their microbiome is poorly understood. At the very least, insect herbivores, like orthopterans, likely rely on their microbiomes to extract nutrients from difficult to digest plant tissues [18]. Therefore, understanding the microbiomes of live-captured grasshoppers from the field offers an opportunity to better understand the functional roles of an important herbivore host in the broader context of their environment. There is a strong possibility that grasshopper species from the same habitat, with similar natural histories, may share a characteristic or “core” group of microbes in their gut. Recent efforts to understand insect herbivore microbiomes have sampled from frass, the gut in sections, or the gut in its entirety [19–21]. In this study, to construct a more complete picture of grasshopper gut bacterial communities, we targeted the microbial community from the entire gut of six different grasshopper species collected from a Texas coastal tallgrass prairie. We identified a core group of common bacterial taxa, including those that contain well-known representatives of plant and animal pathogens, present in all six grasshopper species and detected a significant difference in alpha diversity of bacterial communities present in male versus female grasshoppers.

## Materials and methods

### Sample collection

Grasshoppers were collected using sweep nets (100 sweeps per sampling) from a Texas coastal tallgrass prairie in 2017 within a large-scale fertilization experiment [22]. The grasshoppers were then shipped on ice to the University of Dayton (Dayton, OH) and stored frozen at -20°C until identification and dissection. Grasshopper species and sex were identified and recorded. We used only grasshoppers that could be easily dissected (individuals at 4^th^ instar or later in development). Dissection was performed on each grasshopper by removing the entire gut, including contents, from the crop to the hindgut using instruments sterilized in 95% (v/v) lab-grade ethanol between each dissection.

#### DNA extraction

Each grasshopper gut sample and its contents were first homogenized into smaller pieces inside sterile microcentrifuge tubes using scissors sterilized by 95% lab-grade ethanol and flaming. DNA was extracted using the Qiagen DNeasy Blood and Tissue Kit (Qiagen 69504) following the manufacturer’s protocol with some modifications. Briefly, the length of sample digestion time was increased to 60 minutes in a 57°C water bath, followed by mechanical lysis by bead beating in a Fisherbrand Bead Mill 4 Homogenizer (Fisher Scientific 15-340-152) using 0.5 mm glass beads (Fisher Scientific 15-340-152) for 5 rounds of 60 seconds each at 5 m/s to assure lysis of bacterial cell wall. The bead tubes were then centrifuged for 1 minute at 8,000 rpm to separate lysate from debris. The lysate was placed into a DNeasy collection column for further purification following the manufacturer’s protocol. Following extraction, the concentration of total DNA in each sample was measured by a nanophotometer (Implen, Denville Scientific Inc.). The extracted DNA was stored at -20°C until sequencing.

### 16S rRNA sequencing

High throughput DNA sequencing was performed by Zymo Research (Irvine, CA). DNA from specimens was quantified using a NanoDrop apparatus as well as the 2100 Bioanalyzer system (Agilent) prior to library construction. The sequencing library was prepared by PCR reactions by Zymo Research in real-time PCR machines to control cycles and therefore prevent PCR chimera formation. The V3-V4 region of the 16S rRNA gene was amplified using the Quick-16S Primer Set V3-V4^™^ (Zymo Research, Irvine, CA). The primers used included coverage of both bacteria and archaea. The final PCR products were quantified with qPCR fluorescence readings and pooled together based on equal molarity. The final pooled library was cleaned up with Select-a-Size DNA Clean & Concentrator^™^ (Zymo Research, Irvine, CA), then quantified with TapeStation^®^ and Qubit^®^. DNA libraries from grasshopper gut samples were sequenced on the Illumina HiSeq2500 platform in “Rapid Run” mode with a v3 reagent kit (600 cycles), using 100 bp paired end sequencing, with an average of 10.2 million reads per sample. Samples were collected at various cycles (in 4 cycle intervals) until sufficient amplification had occurred, or at 42 cycles if no amplification occurred (Zymo Research, Irvine, CA). The sequencing was performed with >10% PhiX spike-in [23–25]. DADA2 software, identifying single-nucleotide differences among sequences, was used in concert with the Greengenes database (gg_13_8) to assign bacterial taxa.

### Statistical analysis

Statistical analysis was performed in R (version 3.5.0). After checks for normality indicated non-normal distribution, non-parametric Kruskal-Wallis tests were used for significance calculations. Richness, Shannon diversity, and inverse Simpson diversity calculations were done using the *vegan* package in R [26]. Dominant phyla and families were those that contributed a mean relative abundance of over 1.5% of the sequences for at least one grasshopper species within the taxonomic level being examined. Non-metric multidimensional scaling (NMDS) was performed using the *vegan* package in R. NMDS plots were used if the model arrived at convergence. The *labdsv* package in R was used to calculate indicator groups for each grasshopper species using the indval function in R. Analysis of similarity (ANOSIM) to measure Bray-Curtis dissimilarity, corresponding with the NMDS plots was also performed using the *vegan* in R.

## Results

**Fig 1:**
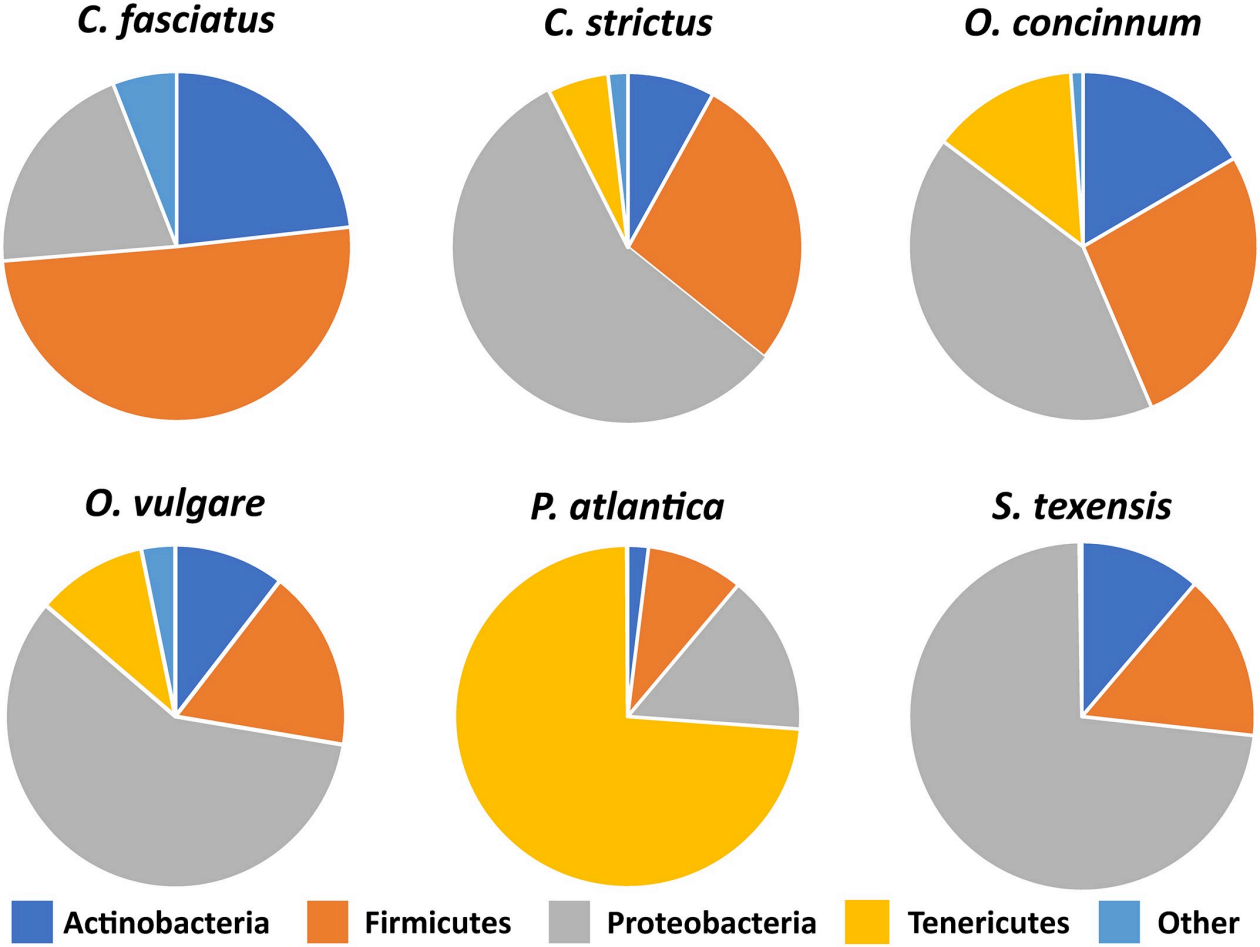
Mean relative abundance of bacterial phyla in the gut of six grasshopper species. Bacterial phyla, predicted from the amplified V3-V4 region of the bacterial 16s rRNA gene, were shown if they were present at >1.5% relative abundance in each grasshopper species. Phyla that were present at <1.5% relative abundance in each grasshopper species are grouped together as “Other.” For specific values, see S2 File.

A total of sixty grasshoppers (Table 1) were sweep-net harvested from the Texas tallgrass coastal prairie, including six different species: *Conocephalus fasciatus* (n = 3), *Conocephalus strictus* (n = 8), *Orchelimum concinnum* (n = 9), *Orchelimum vulgare* (n = 32), *Paroxya atlantica* (n = 4), and *Scudderia texensis* (n = 4). The differing numbers of individuals from each species represent the relative abundance of these grasshopper species in the field. While no archaea were detected, a total of 293 bacterial taxa were obtained from the 60 gut samples. Mean species richness for all six grasshopper species was 27.22 (Table 1). Notably, we found that all six grasshopper species overlapped in their dominant bacterial phyla (Fig 1). However, depending on the grasshopper species, there were shifts in prevalence from one phylum to another. The overall composition of the grasshopper gut bacterial community at the phylum level consisted mainly of *Proteobacteria*, *Actinobacteria*, *Firmicutes*, and to a lesser degree, *Tenericutes* (Fig 1).

**Table 1:** An overview of sample distribution and characterizations.

Species	Sex	n=	Mean Species Richness	Mean Shannon Diversity	Mean Inverse Simpson
All samples	All	60	27.22	1.95	5.18
	Female	30	25.47	1.61	4.18
	Male	30	28.50	2.10	6.37
*Conecephalus faciatus*	All	3	19.67	2.23	10.52
	Female	1	10.00	0.88	1.81
	Male	2	24.50	2.90	14.88
*Conecephalus strictus*	All	8	23.88	1.19	5.46
	Female	3	16.00	1.00	2.12
	Male	5	28.60	2.31	7.46
*Orchelimum concinnum*	All	9	26.44	1.65	4.44
	Female	4	24.40	1.51	4.69
	Male	5	29.00	1.83	4.14
*Orchelimum vulgare*	All	32	30.28	2.00	5.15
	Female	16	31.13	1.86	4.64
	Male	16	29.44	2.13	6.19
*Paroxya atlantica*	All	4	21.50	1.06	2.35
	Female	1	10.00	0.16	1.05
	Male	3	25.33	1.36	2.78
*Scudderia texensis*	All	4	24.33	1.95	5.18
	Female	4	24.33	1.95	5.18
	Male*	-	-	-	-

*No male individuals were collected from the sampling site. For individual grasshopper data, see S1 File.

**Fig 2:**
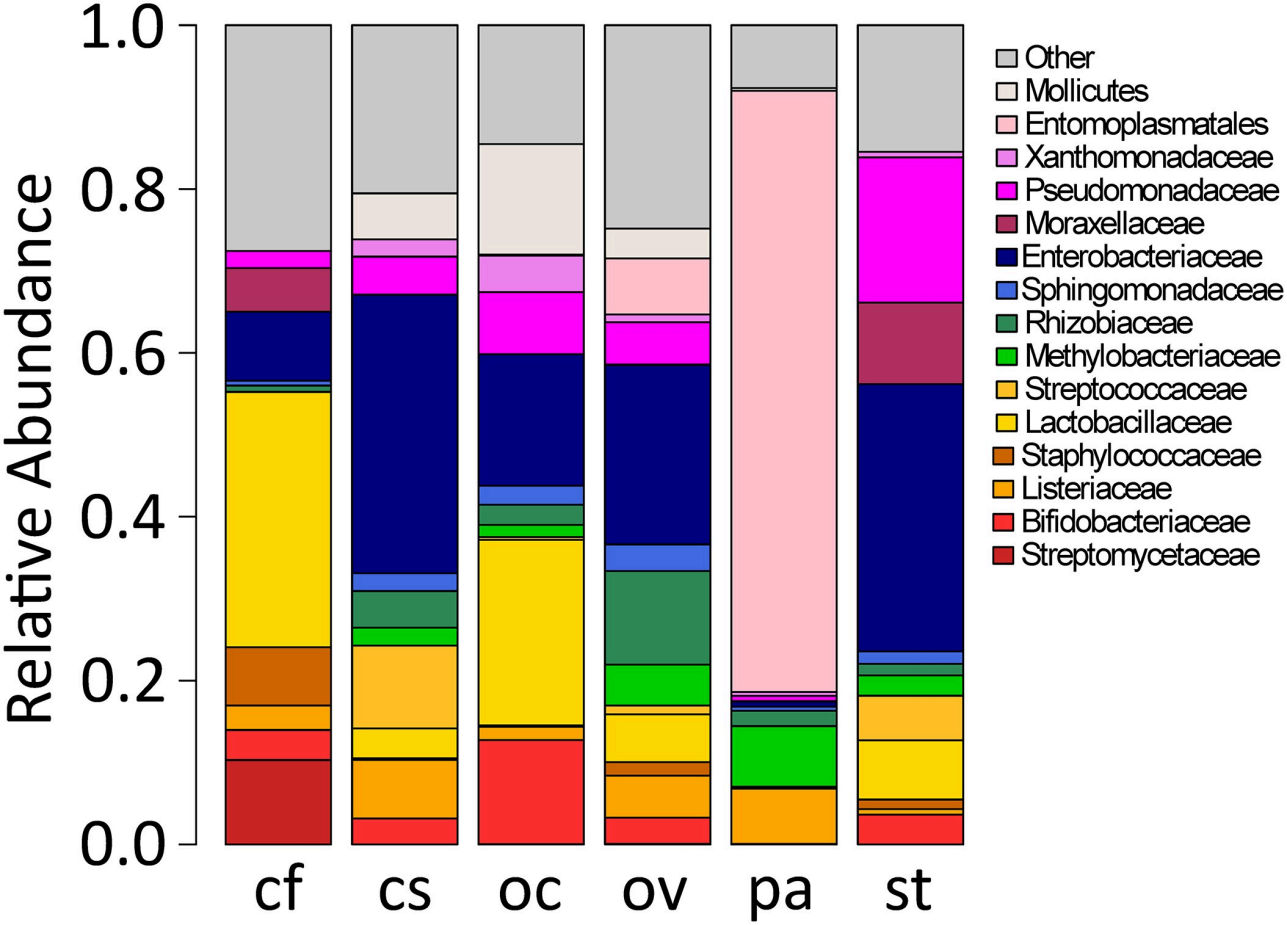
Mean relative abundance of prevalent bacterial families in the gut of six grasshopper species. The abbreviations for grasshopper species that appear on the x axis are as follows: cf, *C*. *fasciatus*; cs, *C*. *strictus*; oc, *O*. *concinnum*; ov, *O*. *vulgare*; pa, *P*. *atlantica*; and st, *S*. *texensis*. Bacterial families with mean relative abundance <1.5% across each grasshopper species are grouped together as “other.” For specific values, see S3 File.

**Fig 3:**
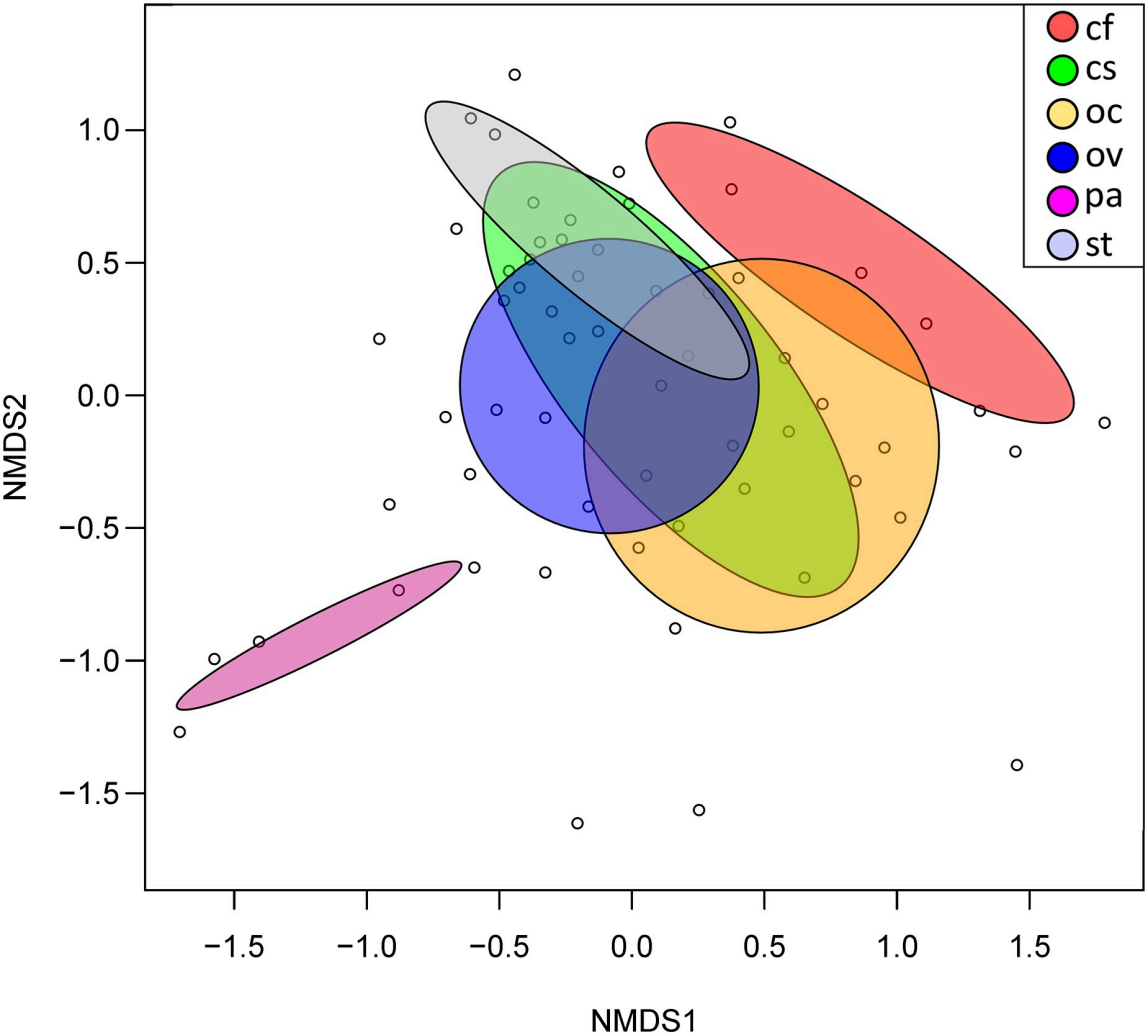
Beta diversity comparison of bacterial communities. Nonmetric multidimensional scaling (stress = 0.084) of grasshopper gut community assemblages at the family level (n = 60) was plotted using the *vegan* package in R. Dispersion ellipses are placed for each grasshopper species. Bray-Curtis dissimilarity was well-preserved in two dimensions. Analysis of Similarity (ANOSIM) was performed with a Bray-Curtis dissimilarity measure, and showed an overall significant difference in gut microbiota among grasshopper species (R = 0.241, p = 0.004).

The most prevalent families included: *Listeriaceae (n = 51 samples)*, *Lactobacillaceae (n = 45)*, *Methylobacteriaceae (n = 47)*, *Rhizobiaceae (n = 50)*, *Sphingomonadaceae (n = 48)*, *Enterobacteriaceae (n = 55)*, and *Pseudomonadaceae (n = 59)* (Fig 2). High abundance sequences also included unassigned families from the order *Entomoplasmatales* and class *Mollicutes*. Analysis of similarity at the level of microbial family indicated a high degree of similarity between the microbiomes of the six species of grasshoppers (Bray-Curtis, R = 0.241, p = 0.004), implying a potential “core” group of microbes that compose the gut microbiome of these insects (Fig 3).

The most notable indicator bacterial group for the six grasshopper species analyzed was the phylum Tenericutes, which was present at relatively low levels in most grasshopper species but clearly dominated the microbiome of *P*. *atlantica* (Fig 1). Indicator group analysis generated a strong indicator value (0.7126, p = 0.006) for Tenericutes in *P*. *atlantica* samples. Non-metric multidimensional scaling (NMDS) performed at the family level (Fig 3) revealed a clear separation between the bacterial communities in *P*. *atlantica* and those in other grasshopper species. The NMDS analysis also shows that despite the shifts in phylum and family prevalence for the other species of grasshopper (Figs 1 and 2), there is a clear overlap in a “core” or characteristic microbiome.

**Fig 4:**
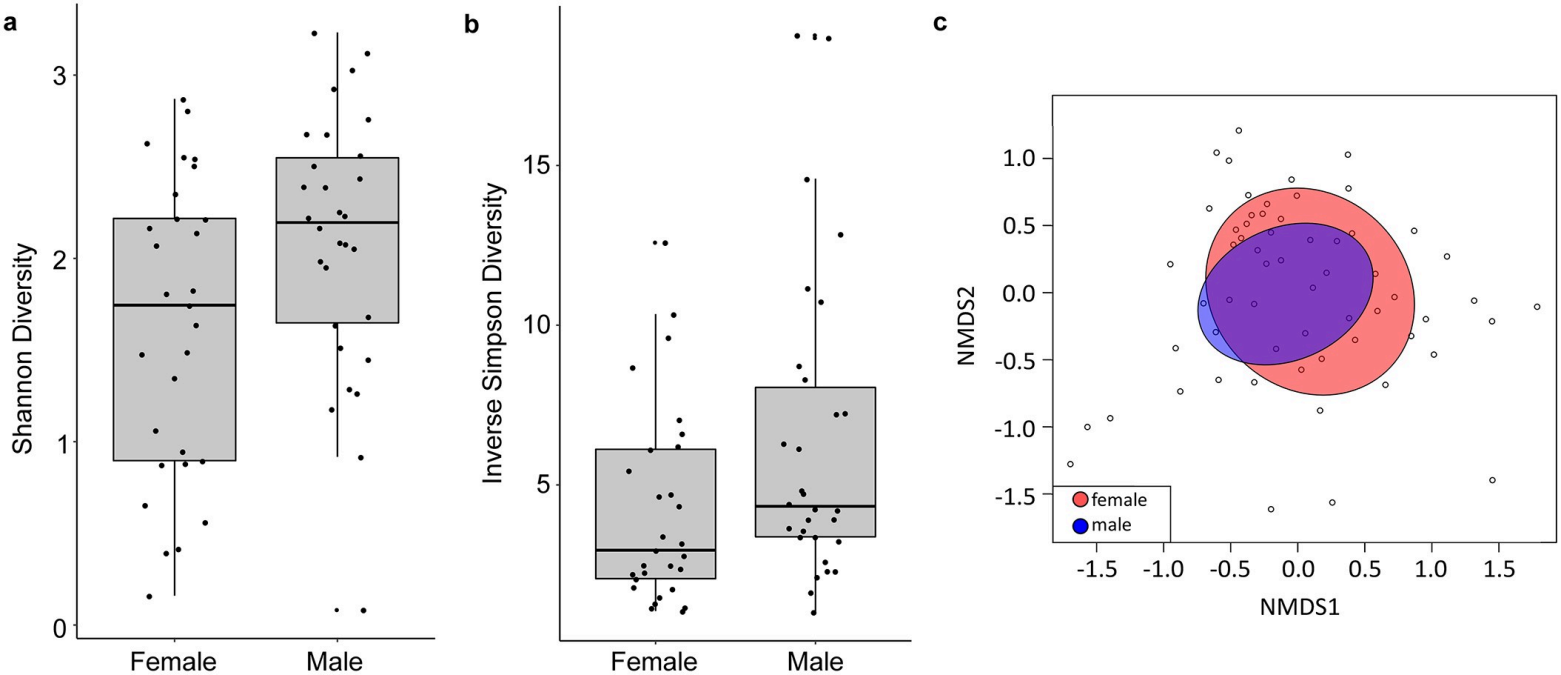
Comparison of the gut microbiome in male and female grasshoppers. Shannon diversity (a) and inverse Simpson diversity (b) indices for each female or male individuals were calculated with the *vegan* package in R. Nonmetric multidimensional scaling with dispersion ellipses placed for male and female pooled species (c) was plotted with the *vegan* package in R. NMDS was constructed using Bray-Curtis dissimilarity. Analysis of Similarity (ANOSIM) was performed and did not show a significant difference in the characteristic gut microbiota of males and females (R = 0.004, p = 0.352).

When males and females of all species were pooled together and compared, there was a significant difference in alpha diversity between males and females with males having higher richness and significantly higher Shannon and inverse Simpson diversity (Fig 4a and 4b; non-parametric Kruskal-Wallis test, p = 0.031 and p = 0.038, respectively). NMDS of the same data showed that although male and female microbiomes overlapped for the most part, female microbiomes were less closely clustered to one another than male microbiomes (Fig 4c). There were strong indicator values for male association with *Listeriaceae* (indicator value = 0.6853, p = 0.017) and *Staphylococcaceae* (indicator value = 0.5326, p = 0.008). These results highlight a previously unrecognized distinction between male and female grasshopper gut microbiomes across different species.

## Discussion

We found that grasshopper guts play host to diverse communities of bacteria. A total of 293 bacterial taxa at the species or strain level were present, with the conservation of Proteobacteria, Actinobacteria, and Firmicutes phyla in all the six grasshopper species. The highest number of taxa present per gut sample in our study was 65, with an average of 26.82 and a median of 27. Although these six grasshopper species do not have the same gut microbiome diversity as mammals, they do have bacterial families similar to other insect herbivores. For example, *Lactobacilliaceae* and *Enterobacteriaceae* families were also found in the guts of Mormon crickets, another orthopteran [19]. Actinobacteria, Firmicutes, and Proteobacteria have all been found in locusts, also orthopterans, [27] and in the well-characterized microbiota of honeybees [15,16,28]. The six species of grasshoppers studied here also overlapped abundant phyla with neotropical butterflies whose dominant groups included Firmicutes, Proteobacteria, and Tenericutes [29]. In contrast, the microbial communities in humans and other mammals tend to be dominated by Bacteroidetes and Firmicutes [30].

The composition of bacterial communities associated with a host organism can be attributed to both an organism’s ecology and evolution [31,32]. Differences in the bacterial community structure among different organisms, therefore, may depend on host species behavior, modes of bacterial community transmission, timing in the lifecycle of the host, evolution, or changes to the environment [33,34]. By analyzing the gut microbiome of 6 different grasshopper species, we have a novel opportunity to examine the correlation between the bacterial communities and the ecology and evolution of their host organisms. The distinctive gut microbiome in *P*. *atlantica* in comparison to other five grasshopper species can be rationalized by the organism’s phylogenetic separation as well as its different dietary preferences. Although commonly referred to as long-horned grasshoppers, *C*. *fasciatus*, *C*. *strictus*, *O*. *concinnum*, and *O*. *vulgare*, are all meadow katydids from the subfamily Conecephalinae. *S*. *texensis* is also a katydid of the subfamily Phaneropterinae, a closely related group to the Conecephalinae [35]. *P*. *atlantica*, on the other hand, is in the subfamily Cyrtacanthacridinae, a short-horned grasshopper. While long-horned grasshoppers look very similar to true grasshoppers, they are more closely related to crickets. Also, the dietary tendency for katydids is omnivory, while short-horned grasshoppers, like *P*. *atlantica*, tend to eat almost exclusively plant matter. The structure of the tooth mandible in *P*. *atlantica* suggests that this species specializes on forbs, but a tendency to eat grasses has also been experimentally verified [36]. Therefore, the distinctive bacterial communities associated with *P*. *atlantica*, in comparison to the other five species, can be supported by the phylogenetic relationship or the dietary preferences. A wider investigation into the gut microbiome in additional grasshopper species in the future could help establish whether there are evolutionary associations between the phylogeny of different grasshopper species and their microbial symbionts or are there simply differences in dietary preferences that shape the grasshopper microbiome.

It is interesting to note that the shift in bacterial prevalence for *P*. *atlantica* is towards the phylum Tenericutes, which has a high indicator value when in association with *P*. *atlantica*. Tenericutes are a phylum of bacteria with small genomes and no cell wall [37]. They are known to have symbiotic relationships with arthropods as well as other animals and plants [37–40]. Within the phylum Tenericutes is the class Mollicutes, which includes the order Entomoplasmatales that houses many species which have widely reported associations with insects, including spiroplasmas [38,39]. Although the bacterial taxa associated with the Mollicutes did not resolve to specific families in our sequence analysis, multiple unique, unclassified Entomoplasmatales family reads were present, indicating the possibility of spiroplasmas in the grasshopper gut. It remains to be seen whether there is a significant host-microbe relationship for *P*. *atlantica* and a specific spiroplasma species. In some cases, spiroplasmas are harmful, disease-causing pathogens, and in other cases, vertically-transferred symbionts [38]. The absence of spiroplasmas in pea-aphids affects host fitness by decreasing offspring production and survival [39]. Other known Entomoplasmatales-insect associations include *Dabulus maidis* leafhoppers, Heliconius butterflies, New World army ants, and *Drosophila hydei*, which have been shown to have nonpathogenic entomoplasmatales in their gut that may be linked to increased host survival and fitness [38–43]. Finally, in mammals, mice fed a high-fat diet have decreased numbers of Tenericutes in their gut, suggesting that there is a possible connection between diet in mammals and levels of Tenericutes [44]. At this point, the relationship of *P*. *atlantica* with Entomoplasmales in particular, or Tenericutes in general, is unknown. It is also not known whether the relationship is evolutionary or dietary in origin. Both rationales are possible given both *P*. *atlantica*’s phylogenetic distance from the other grasshoppers in this study and its difference in trophic ecology [45].

Male grasshoppers in our study showed a significantly higher alpha diversity (Fig 4a and 4b). Because the prevalence of the main phyla does not shift between male and female (Fig 4c), it is possible that the differences between male and female grasshopper gut microbiota could be attributed to rare taxa. This is true for some species of butterflies, who despite having no significant differences in alpha diversity between males and females, still have different rare taxa [43]. However, given that the overlap between male and female grasshopper microbiome is not complete, it is also possible that there are subtle shifts in main taxa groups that account for the difference in alpha diversity between males and females. Functionally, it is unclear what drives the sexual dimorphism in the grasshopper microbiome, but potential roles have been reported in other animals. In mice, the gut microbiome is integral to the sexual dimorphism in the regulation of hormones for growth and sexual maturity, as well as improving disease resistance [46,47]. Female *Drosophila melanogaster* mating patterns are correlated with male microbiota so that females mate preferentially with males with intact microbiota [48]. In the moth, *Spodoptera littoralis*, adult female microbiomes contain a higher number of genes for energy metabolism than male microbiomes [5]. Future studies that further dissect potential sexual dimorphism in the insect gut microbiome will likely reveal additional functional relationships between insects and their resident microbes.

All the prevalent families found in the grasshopper guts (Fig 2) appear to have members with well-known associations with soils and plants that may contribute to their associations with grasshoppers. Understandably, the presence of these taxa, as established by DNA sequencing, is not a direct indication of functions or permanent residency. Nevertheless, it is of interest to note that some of these bacterial families may have a functional role to play in the gut, while others may be plant or insect pathogens for which the grasshopper may serve as a reservoir or vector [4,49]. For example, Enterobacteriaceae family was found in almost every gut sample analyzed. Insect symbionts from this family have been found to counteract plant defenses and insecticides [50]. *Erwinia*, which has been identified as a probable symbiont of western flower thrips [51], was also present in our samples. Another family with relatively high abundance was Lactobacilliaceae, which has been reported extensively in honeybees. The microbiome composition in honey bees appear to correlate with behavioral tasks such as foraging, caring for young, or food processing—with *Lactobacillus mellis* present at a significantly higher level in bees performing nursing or processing food than bees performing foraging tasks [52,53]. The bee-associated *Lactobacillus* species (Family Lactobacillaceae) are known to be phylogenetically and metabolically distinct from human-associated *Lactobacillus* species [54]. Similarly, *Bifidobacterium* (Family Bifidobacteriaceae) associated with humans and monkeys also possess higher levels of glycosyl hydrolases than those from insects or other animals and environment [54]. These observations, while supporting the metabolic roles of gut microbiome in host behavior and nutrient acquisition, also highlight the need to better understand the evolutionary history behind the association of animals, from grasshoppers to mammals, with these common bacterial families. It is also worth noting the presence of potential plant and animal pathogens within the grasshopper gut microbiome. This fact raises questions on the role of grasshoppers in disease transmission. Families Pseudomonadaceae and Xanthomonadaceae both have members that are highly critical plant pathogens [55]. Family Methylobacteriaceae, present in 75 percent of grasshoppers sampled, includes *Methylobacterium* species that are typically found in soil and water that may be transiently associated with grasshoppers. However, despite its typical free-living lifestyle, one species, *Methylobacterium mesophilicum*, has a demonstrated ability to stably colonize *Catharanthus roseus*, or Madagascar periwinkle, through the transmission by the insect *Bucephalogonia xanthophis* (Hemiptera: Cicadellidae) [56]. Most known plant pathogens are transmitted by Hemipteran insects [57]; however, if grasshoppers prove to be carriers of plant pathogens, even transiently, they may be able to contribute to disease transmission and undermine efforts to protect crops by sustaining the pathogens in the environment.

The relatively high prevalence of the family Listeriaceae, especially among male grasshoppers (indicator value = 0.6853, p = 0.017), observed in this study has not been routinely acknowledged in any insects. The family Listeriaceae includes two genera: *Listeria* and *Brochothrix* [58], of which several species of *Listeria* are known human and animal pathogens with others containing pathogenicity-associated genes [59]. Therefore, for the interest of food safety, the presence of *Listeria* in insects, especially potentially edible insects, has been an ongoing investigation. In numerous other surveys of insects, including bush crickets (*Ruspolia diffferens*), lesser mealworms (*Alphitobius diaperinus*), tropical house crickets (*Gryllodes sigillatus*), field crickets (*Gryllus bimaculatus*), superworms (*Zophobas atratus*), and cockroaches, no *Listeria* has been found [60–64]. This absence of *Listeria* in insects, despite the prevalence of *Listeria* in natural environments [65], might result from *Listeria* acting as an insect pathogen. Multiple insects, including greater wax moth (*Galleria mellonella*), 2-spotted cricket (*Gryllus bimaculatus*), house fly (*Musca domestica*), and common fruit fly (*Drosophila melanogaster*), have all been suggested as host models to study *Listeria* infections [65–68]. Curiously, *Listeria* has been cultured from ants collected from Pamplemousses, Mauritius, as well as from citrus black flies (*Aleurocanthus woglumi* Ashby) [69,70], suggesting that some insects, perhaps grasshoppers included, are capable of establishing a symbiotic relationship with *Listeria* species. Whether grasshoppers from a coastal prairie system serve as an environmental reservoir for potential human pathogens remains to be determined. However, there might be an association between *Listeria* and certain insects that contribute to the ubiquity of *Listeria* in the environment.

## Conclusion

In this study, we demonstrated the conservation of three phylum, Actinobacteria, Proteobacteria, and Firmicutes across six different species of grasshoppers. A fourth bacterial phylum, Tenericutes, is present in four of the six grasshopper species and dominates the microbiome of the short-horned grasshopper herbivore, *P*. *atlantica*. This core microbiome overlaps with the microbiome found in many other insect herbivores. Differences in the bacterial community compositions in the six species of grasshoppers may be influenced by evolutionary mechanisms or dietary preferences. While there are consistent bacterial groups across all six species, the function of these host microbiomes and their influence on the ecosystem remains an open question. A deeper understanding of these important host-microbiome interactions will provide insight into both host ecology and bacterial functions that may ultimately lead to better conservation of these animals and their habitats and more safe and effective pest control measures.

## Supporting information

S1 File Diversity indices for individual grasshoppers. (CSV)

S2 File Average relative abundance of bacterial phyla. (CSV)

S3 File Average relative abundance of bacterial families. (CSV)
